# 3-(5,6,7,8-Tetra­hydro-2-naphth­yl)iso­benzofuran-1(3*H*)-one

**DOI:** 10.1107/S1600536808024598

**Published:** 2008-08-06

**Authors:** Chitoshi Kitamura, Takeshi Kawase

**Affiliations:** a Department of Materials Science and Chemistry, Graduate School of Engineering, University of Hyogo, 2167 Shosha, Himeji, Hyogo 671-2280, Japan

## Abstract

The title compound, C_18_H_16_O_2_, was prepared by reduction of 2-(5,6,7,8-tetra­hydro-2-naphtho­yl)benzoic acid with zinc dust. The benzene ring in the tetra­hydro­naphthyl substituent is nearly perpendicular to the plane of the isobenzofuran-1(3*H*)-one ring [87.15 (4)°]. The cyclo­hexane unit has a half-chair conformation in which two methylene groups in the tetra­methyl­ene bridge are disordered over two positions; the site-occupancy factors are 0.838 (4) and 0.162 (4). The crystal structure exhibits alternating isobenzofuran-1(3*H*)-one and tetra­hydro­naphthalene layers.

## Related literature

For related mol­ecular structures, including a 3-phenyl isobenzofuran-1(3*H*)-one system, see: Chan & Scheidt (2006 ▶); Kalyani & Vijayan (1969 ▶); Vijayan *et al.* (2006 ▶). For related literature, see: Konosonoks *et al.* (2005 ▶); Schroeter (1921 ▶).
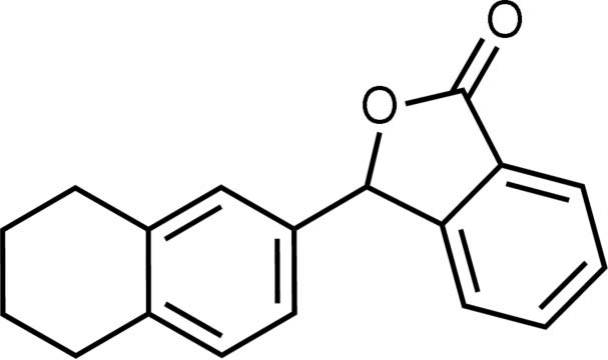


## Experimental

### 

#### Crystal data

C_18_H_16_O_2_
*M* *_r_* = 264.31
Monoclinic, 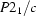

*a* = 11.2950 (11) Å
*b* = 15.8251 (10) Å
*c* = 7.8092 (10) Å
β = 109.0970 (10)°
*V* = 1319.0 (2) Å^3^
*Z* = 4
Mo *K*α radiation
μ = 0.09 mm^−1^
*T* = 223 K
0.5 × 0.5 × 0.03 mm

#### Data collection

Rigaku/MSC Mercury CCD area-detector diffractometer
Absorption correction: numerical (*NUMABS*; Higashi, 1999 ▶) *T*_min_ = 0.980, *T*_max_ = 0.995
5765 measured reflections
2955 independent reflections
2502 reflections with *I* > 2σ(*I*)
*R*_int_ = 0.015

#### Refinement

*R*[*F* ^2^ > 2σ(*F* ^2^)] = 0.038
*wR*(*F* ^2^) = 0.108
*S* = 1.1
2955 reflections
200 parameters
3 restraints
H-atom parameters constrained
Δρ_max_ = 0.23 e Å^−3^
Δρ_min_ = −0.16 e Å^−3^

### 

Data collection: *CrystalClear* (Rigaku/MSC, 2001 ▶); cell refinement: *CrystalClear*; data reduction: *CrystalClear* and *WinGX* (Farrugia, 1999 ▶); program(s) used to solve structure: *SIR2004* (Burla *et al.*, 2005 ▶); program(s) used to refine structure: *SHELXL97* (Sheldrick, 2008 ▶); molecular graphics: *ORTEP-3 for Windows* (Farrugia, 1997 ▶); software used to prepare material for publication: *WinGX*.

## Supplementary Material

Crystal structure: contains datablocks global, I. DOI: 10.1107/S1600536808024598/pk2111sup1.cif

Structure factors: contains datablocks I. DOI: 10.1107/S1600536808024598/pk2111Isup2.hkl

Additional supplementary materials:  crystallographic information; 3D view; checkCIF report

## Figures and Tables

**Fig. 1.**
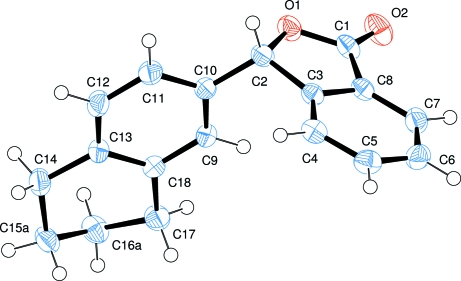
The molecular structure of (I), showing 50% probability displacement ellipsoids for non-H atoms. The minor occupied site of the disordered methylene-bridge chain is omitted for clarity.

**Fig. 2.**
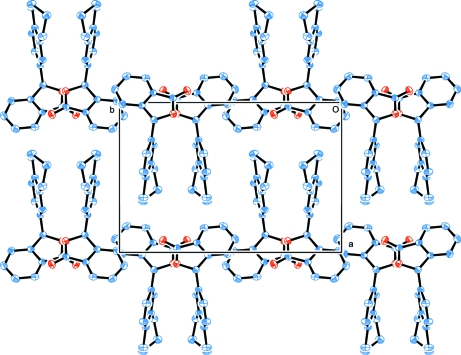
The packing diagram of (I), viewed down the c axis. Hydrogen atoms are omitted for clarity.

## supplementary crystallographic information

### Comment

A number of 3-phenylisobenzofuran-1(3*H*)-one derivatives have been prepared from the corresponding 2-benzoylbenzoic acid by reduction using zinc dust. Several crystal structures including 3-phenylisobenzofuran-1(3*H*)-one were reported (Chan & Scheidt, 2006; Kalyani & Vijayan, 1969; Konosonoks *et al.*, 2005; Vijayan *et al.*, 2006). The title compound, which was first prepared by Schroeter (1921), can be regarded as a derivative of 3-phenylisobenzofuran-1(3*H*)-one by annelation of cyclohexane to the substituent phenyl ring. In order to ascertain the effect of the annelation of cyclohexane into the structure, X-ray analysis was performed.

The molecular structure is shown in Fig. 1. The isobenzofuran-1(3*H*)-one moiety is essentially planar. The benzene ring within the tetrahydronaphthyl substituent is nearly perpendicular to the plane of the isobenzofuran-1(3*H*)-one ring (87.13 (3)°). The dihedral angle O1—C2—C10—C9 between the isobenzofuran-1(3*H*)-one ring and the benzene ring is 58.55 (12)°, and is smaller than that (64.49°) of the corresponding 3-phenylisobenzofuran-1(3*H*)-one (Chan & Scheidt, 2006). The annelated cyclohexane ring has a half-chair configuration. The ethylene unit in the tetramethylene-bridge is disordered over two sites (C14—C15A—C16A—C17 and C14—C15B—C16B—C17) with refined occupancies of 0.838 (4) and 0.162 (4).

As shown in Fig. 2, the crystal structure is characterized by two alternating layers, which consist of the isobenzofuran-1(3*H*)-one layer lying on the *bc* planes and the tetrahydro naphthalene layer, which exists between the *bc* planes.

### Experimental

The title compound was prepared according to the modified method described by Schroeter (1921). A mixture of 2-(5,6,7,8-tetrahydronaphtho-2-yl)benzoic acid (2.01 g, 7.16 mmol) and zinc dust (2.00 g, 30.6 mmol) in 25% ammonia solution (30 ml) was refluxed for 4 h. The reaction mixture was cooled and conc. HCl (8 ml) was added. The resulting precipitate was filtered off, and washed with dichloromethane. The organic layer was separated, washed with brine, and dried over Na_2_SO_4_. After evaporation, column chromatography on silica gel (CH_2_Cl_2_) gave the compound (1.17 g, 62%) as a white solid. Colourless crystals suitable for X-ray analysis were obtained from a dichloromethane solution.

### Refinement

All the H atoms were positioned geometrically and refined using a riding model with C_aromatic_—H = 0.94Å [*U*_iso_(H) = 1.2*U*_eq_(C)] and C_methylene_—H = 0.98Å [*U*_iso_(H) = 1.2*U*_eq_(C)]. The methylene carbon atoms (C15A, C15B, C16A, and C16B) and the associated hydrogen atoms are disordered over two sites (C14—C15A—C16A—C17 and C14—C15B—C16B—C17) with occupancies of 0.838 (4) and 0.162 (4). The values were determined by refining site occupancies. Three C—C distances (C14—C15B, C15B—C16B, and C16B—C17) of the disordered atoms (C15B and C16B) were restrained to 1.54 (1) Å.

### Crystal data

**Table:**

C_18_H_16_O_2_	*F*_000_ = 560
*M**_r_* = 264.31	*D*_x_ = 1.331 Mg m^−^^3^
Monoclinic, *P*2_1_/*c*	Mo *K*α radiation λ = 0.71073 Å
Hall symbol: -P 2ybc	Cell parameters from 4161 reflections
*a* = 11.2950 (11) Å	θ = 3.1–27.5º
*b* = 15.8251 (10) Å	µ = 0.09 mm^−^^1^
*c* = 7.8092 (10) Å	*T* = 223 K
β = 109.0970 (10)º	Platelet, colourless
*V* = 1319.0 (2) Å^3^	0.5 × 0.5 × 0.03 mm
*Z* = 4	

### Data collection

**Table:**

Rigaku/MSC Mercury CCD area-detector diffractometer	2955 independent reflections
Radiation source: rotating-anode X-ray tube	2502 reflections with *I* > 2σ(*I*)
Monochromator: graphite	*R*_int_ = 0.015
Detector resolution: 14.7059 pixels mm^-1^	θ_max_ = 27.5º
*T* = 223 K	θ_min_ = 3.1º
φ and ω scans	*h* = −14→0
Absorption correction: numerical(NUMABS; Higashi, 1999)	*k* = −20→20
*T*_min_ = 0.980, *T*_max_ = 0.995	*l* = −9→10
5765 measured reflections	

### Refinement

**Table:**

Refinement on *F*^2^	Secondary atom site location: difference Fourier map
Least-squares matrix: full	Hydrogen site location: inferred from neighbouring sites
*R*[*F*^2^ > 2σ(*F*^2^)] = 0.038	H-atom parameters constrained
*wR*(*F*^2^) = 0.108	*w* = 1/[σ^2^(*F*_o_^2^) + (0.1123*P*)^2^ + 0.367*P*] where *P* = (*F*_o_^2^ + 2*F*_c_^2^)/3
*S* = 1.1	(Δ/σ)_max_ = 0.001
2955 reflections	Δρ_max_ = 0.23 e Å^−^^3^
200 parameters	Δρ_min_ = −0.16 e Å^−^^3^
3 restraints	Extinction correction: none
Primary atom site location: structure-invariant direct methods	

### Special details

**Table:**

Geometry. All e.s.d.'s (except the e.s.d. in the dihedral angle between two l.s. planes) are estimated using the full covariance matrix. The cell e.s.d.'s are taken into account individually in the estimation of e.s.d.'s in distances, angles and torsion angles; correlations between e.s.d.'s in cell parameters are only used when they are defined by crystal symmetry. An approximate (isotropic) treatment of cell e.s.d.'s is used for estimating e.s.d.'s involving l.s. planes.
Refinement. Refinement of *F*^2^ against ALL reflections. The weighted *R*-factor *wR* and goodness of fit *S* are based on *F*^2^, conventional *R*-factors *R* are based on *F*, with *F* set to zero for negative *F*^2^. The threshold expression of *F*^2^ > σ(*F*^2^) is used only for calculating *R*-factors(gt) *etc*. and is not relevant to the choice of reflections for refinement. *R*-factors based on *F*^2^ are statistically about twice as large as those based on *F*, and *R*- factors based on ALL data will be even larger.

### Fractional atomic coordinates and isotropic or equivalent isotropic displacement parameters (Å^2^)

**Table:**

	*x*	*y*	*z*	*U*_iso_*/*U*_eq_	Occ. (<1)
C1	1.02994 (10)	0.24031 (7)	0.24158 (14)	0.0297 (2)	
C2	0.88406 (10)	0.15608 (7)	0.31444 (13)	0.0275 (2)	
H2	0.8899	0.1481	0.4427	0.033*	
C3	0.97929 (9)	0.10068 (7)	0.27234 (12)	0.0253 (2)	
C4	0.99036 (10)	0.01332 (7)	0.27188 (13)	0.0298 (2)	
H4	0.9325	−0.0218	0.3009	0.036*	
C5	1.08967 (11)	−0.02030 (7)	0.22709 (14)	0.0333 (3)	
H5	1.0999	−0.0793	0.2274	0.04*	
C6	1.17465 (11)	0.03146 (8)	0.18158 (14)	0.0349 (3)	
H6	1.241	0.0069	0.1514	0.042*	
C7	1.16291 (10)	0.11850 (7)	0.18020 (14)	0.0314 (2)	
H7	1.2193	0.1537	0.1481	0.038*	
C8	1.06447 (10)	0.15181 (7)	0.22818 (12)	0.0261 (2)	
C9	0.71587 (10)	0.15338 (7)	0.00822 (13)	0.0268 (2)	
H9	0.7772	0.1683	−0.0437	0.032*	
C10	0.75057 (10)	0.14339 (6)	0.19540 (13)	0.0254 (2)	
C11	0.65959 (11)	0.12144 (7)	0.27084 (14)	0.0309 (2)	
H11	0.6811	0.1149	0.397	0.037*	
C12	0.53701 (11)	0.10919 (8)	0.16038 (14)	0.0333 (3)	
H12	0.4763	0.0939	0.2131	0.04*	
C13	0.50144 (10)	0.11891 (6)	−0.02704 (13)	0.0272 (2)	
C14	0.36564 (10)	0.10711 (8)	−0.14102 (15)	0.0368 (3)	
H14A	0.3166	0.1534	−0.1148	0.044*	
H14B	0.3353	0.0543	−0.1046	0.044*	
C15A	0.34138 (19)	0.10439 (13)	−0.3451 (2)	0.0362 (5)	0.838 (4)
H15A	0.3638	0.0486	−0.3796	0.043*	0.838 (4)
H15B	0.2522	0.1141	−0.4099	0.043*	0.838 (4)
C16A	0.41910 (14)	0.17205 (11)	−0.39664 (18)	0.0363 (5)	0.838 (4)
H16A	0.3992	0.2275	−0.3573	0.044*	0.838 (4)
H16B	0.3988	0.1734	−0.5286	0.044*	0.838 (4)
C15B	0.3475 (11)	0.1453 (9)	−0.3284 (11)	0.070 (5)	0.162 (4)
H15C	0.2625	0.1325	−0.4085	0.084*	0.162 (4)
H15D	0.3555	0.2068	−0.3169	0.084*	0.162 (4)
C16B	0.4407 (7)	0.1126 (8)	−0.4145 (10)	0.056 (3)	0.162 (4)
H16C	0.4475	0.0509	−0.4059	0.068*	0.162 (4)
H16D	0.4168	0.1292	−0.5422	0.068*	0.162 (4)
C17	0.55921 (11)	0.15332 (8)	−0.30668 (14)	0.0347 (3)	
H17A	0.5809	0.1019	−0.3597	0.042*	
H17B	0.6084	0.2	−0.3311	0.042*	
C18	0.59290 (10)	0.14175 (6)	−0.10368 (13)	0.0257 (2)	
O1	0.92438 (7)	0.24194 (5)	0.29001 (10)	0.0330 (2)	
O2	1.07896 (8)	0.30458 (5)	0.21603 (11)	0.0418 (2)	

### Atomic displacement parameters (Å^2^)

**Table:**

	*U*^11^	*U*^22^	*U*^33^	*U*^12^	*U*^13^	*U*^23^
C1	0.0239 (5)	0.0292 (6)	0.0300 (5)	−0.0028 (4)	0.0004 (4)	−0.0003 (4)
C2	0.0265 (6)	0.0272 (5)	0.0274 (5)	−0.0017 (4)	0.0067 (4)	−0.0023 (4)
C3	0.0231 (5)	0.0276 (5)	0.0218 (4)	0.0000 (4)	0.0026 (4)	0.0002 (3)
C4	0.0323 (6)	0.0273 (5)	0.0273 (5)	−0.0015 (4)	0.0062 (4)	0.0024 (4)
C5	0.0372 (6)	0.0272 (6)	0.0317 (5)	0.0053 (4)	0.0063 (4)	−0.0004 (4)
C6	0.0300 (6)	0.0401 (6)	0.0334 (5)	0.0072 (5)	0.0088 (4)	−0.0026 (4)
C7	0.0249 (5)	0.0389 (6)	0.0293 (5)	−0.0016 (4)	0.0072 (4)	0.0012 (4)
C8	0.0232 (5)	0.0268 (5)	0.0237 (4)	−0.0011 (4)	0.0014 (4)	0.0004 (3)
C9	0.0247 (5)	0.0297 (5)	0.0284 (5)	−0.0011 (4)	0.0120 (4)	−0.0014 (4)
C10	0.0237 (5)	0.0247 (5)	0.0278 (5)	0.0014 (4)	0.0083 (4)	−0.0023 (4)
C11	0.0309 (6)	0.0385 (6)	0.0241 (5)	−0.0007 (4)	0.0103 (4)	0.0015 (4)
C12	0.0281 (6)	0.0440 (7)	0.0315 (5)	−0.0030 (5)	0.0147 (4)	0.0018 (4)
C13	0.0228 (5)	0.0295 (5)	0.0298 (5)	0.0009 (4)	0.0092 (4)	−0.0003 (4)
C14	0.0240 (6)	0.0509 (7)	0.0349 (6)	−0.0017 (5)	0.0089 (4)	−0.0013 (5)
C15A	0.0275 (8)	0.0474 (11)	0.0288 (8)	−0.0073 (8)	0.0024 (6)	−0.0018 (7)
C16A	0.0345 (9)	0.0412 (10)	0.0286 (7)	−0.0015 (7)	0.0040 (6)	0.0061 (6)
C15B	0.020 (5)	0.123 (13)	0.054 (6)	−0.001 (7)	−0.007 (4)	−0.044 (8)
C16B	0.042 (5)	0.098 (10)	0.027 (4)	0.012 (5)	0.008 (3)	−0.008 (4)
C17	0.0348 (6)	0.0437 (7)	0.0258 (5)	−0.0052 (5)	0.0103 (4)	0.0010 (4)
C18	0.0262 (5)	0.0261 (5)	0.0256 (5)	0.0008 (4)	0.0099 (4)	−0.0007 (4)
O1	0.0272 (4)	0.0260 (4)	0.0430 (4)	−0.0011 (3)	0.0077 (3)	−0.0056 (3)
O2	0.0370 (5)	0.0279 (4)	0.0544 (5)	−0.0074 (3)	0.0066 (4)	0.0031 (3)

### Geometric parameters (Å, °)

**Table:**

C1—O2	1.2054 (13)	C12—C13	1.3937 (14)
C1—O1	1.3639 (14)	C12—H12	0.94
C1—C8	1.4668 (15)	C13—C18	1.4002 (14)
C2—O1	1.4651 (13)	C13—C14	1.5122 (15)
C2—C10	1.5037 (14)	C14—C15A	1.527 (2)
C2—C3	1.5050 (14)	C14—C15B	1.534 (9)
C2—H2	0.99	C14—H14A	0.98
C3—C8	1.3844 (14)	C14—H14B	0.98
C3—C4	1.3881 (15)	C15A—C16A	1.520 (2)
C4—C5	1.3858 (15)	C15A—H15A	0.98
C4—H4	0.94	C15A—H15B	0.98
C5—C6	1.3935 (17)	C16A—C17	1.5357 (19)
C5—H5	0.94	C16A—H16A	0.98
C6—C7	1.3834 (16)	C16A—H16B	0.98
C6—H6	0.94	C15B—C16B	1.514 (9)
C7—C8	1.3882 (15)	C15B—H15C	0.98
C7—H7	0.94	C15B—H15D	0.98
C9—C18	1.3901 (15)	C16B—C17	1.476 (7)
C9—C10	1.3930 (14)	C16B—H16C	0.98
C9—H9	0.94	C16B—H16D	0.98
C10—C11	1.3856 (14)	C17—C18	1.5155 (14)
C11—C12	1.3846 (16)	C17—H17A	0.98
C11—H11	0.94	C17—H17B	0.98
			
O2—C1—O1	121.36 (10)	C13—C14—H14A	108.6
O2—C1—C8	130.29 (11)	C15A—C14—H14A	108.6
O1—C1—C8	108.35 (9)	C15B—C14—H14A	89.8
O1—C2—C10	109.57 (8)	C13—C14—H14B	108.6
O1—C2—C3	103.71 (8)	C15A—C14—H14B	108.6
C10—C2—C3	115.52 (8)	C15B—C14—H14B	131.5
O1—C2—H2	109.3	H14A—C14—H14B	107.6
C10—C2—H2	109.3	C16A—C15A—C14	109.54 (14)
C3—C2—H2	109.3	C16A—C15A—H15A	109.8
C8—C3—C4	120.72 (10)	C14—C15A—H15A	109.8
C8—C3—C2	108.57 (9)	C16A—C15A—H15B	109.8
C4—C3—C2	130.71 (10)	C14—C15A—H15B	109.8
C5—C4—C3	117.65 (10)	H15A—C15A—H15B	108.2
C5—C4—H4	121.2	C15A—C16A—C17	109.93 (13)
C3—C4—H4	121.2	C15A—C16A—H16A	109.7
C4—C5—C6	121.37 (10)	C17—C16A—H16A	109.7
C4—C5—H5	119.3	C15A—C16A—H16B	109.7
C6—C5—H5	119.3	C17—C16A—H16B	109.7
C7—C6—C5	120.99 (10)	H16A—C16A—H16B	108.2
C7—C6—H6	119.5	C16B—C15B—C14	113.2 (8)
C5—C6—H6	119.5	C16B—C15B—H15C	108.9
C6—C7—C8	117.33 (10)	C14—C15B—H15C	108.9
C6—C7—H7	121.3	C16B—C15B—H15D	108.9
C8—C7—H7	121.3	C14—C15B—H15D	108.9
C3—C8—C7	121.91 (10)	H15C—C15B—H15D	107.7
C3—C8—C1	108.52 (9)	C17—C16B—C15B	103.3 (8)
C7—C8—C1	129.57 (10)	C17—C16B—H16C	111.1
C18—C9—C10	121.66 (9)	C15B—C16B—H16C	111.1
C18—C9—H9	119.2	C17—C16B—H16D	111.1
C10—C9—H9	119.2	C15B—C16B—H16D	111.1
C11—C10—C9	118.78 (9)	H16C—C16B—H16D	109.1
C11—C10—C2	120.25 (9)	C16B—C17—C18	114.5 (4)
C9—C10—C2	120.97 (9)	C18—C17—C16A	111.74 (10)
C12—C11—C10	119.99 (9)	C16B—C17—H17A	72.7
C12—C11—H11	120	C18—C17—H17A	109.3
C10—C11—H11	120	C16A—C17—H17A	109.3
C11—C12—C13	121.65 (10)	C16B—C17—H17B	133.2
C11—C12—H12	119.2	C18—C17—H17B	109.3
C13—C12—H12	119.2	C16A—C17—H17B	109.3
C12—C13—C18	118.55 (9)	H17A—C17—H17B	107.9
C12—C13—C14	119.53 (9)	C9—C18—C13	119.38 (9)
C18—C13—C14	121.90 (9)	C9—C18—C17	119.92 (9)
C13—C14—C15A	114.77 (11)	C13—C18—C17	120.69 (9)
C13—C14—C15B	107.8 (5)	C1—O1—C2	110.84 (8)
			
O1—C2—C3—C8	−0.12 (10)	C11—C12—C13—C14	−178.35 (11)
C10—C2—C3—C8	119.79 (9)	C12—C13—C14—C15A	−170.05 (12)
O1—C2—C3—C4	−179.84 (9)	C18—C13—C14—C15A	11.73 (17)
C10—C2—C3—C4	−59.94 (14)	C12—C13—C14—C15B	164.3 (5)
C8—C3—C4—C5	0.44 (14)	C18—C13—C14—C15B	−13.9 (5)
C2—C3—C4—C5	−179.87 (10)	C13—C14—C15A—C16A	−42.2 (2)
C3—C4—C5—C6	−0.87 (15)	C15B—C14—C15A—C16A	36.6 (10)
C4—C5—C6—C7	0.23 (16)	C14—C15A—C16A—C17	63.7 (2)
C5—C6—C7—C8	0.84 (15)	C13—C14—C15B—C16B	52.2 (11)
C4—C3—C8—C7	0.66 (14)	C15A—C14—C15B—C16B	−58.4 (9)
C2—C3—C8—C7	−179.10 (9)	C14—C15B—C16B—C17	−72.0 (13)
C4—C3—C8—C1	−179.57 (8)	C15B—C16B—C17—C18	52.0 (9)
C2—C3—C8—C1	0.67 (10)	C15B—C16B—C17—C16A	−42.7 (6)
C6—C7—C8—C3	−1.29 (14)	C15A—C16A—C17—C16B	48.9 (5)
C6—C7—C8—C1	178.99 (10)	C15A—C16A—C17—C18	−53.52 (17)
O2—C1—C8—C3	179.48 (11)	C10—C9—C18—C13	−0.41 (15)
O1—C1—C8—C3	−1.02 (10)	C10—C9—C18—C17	179.96 (10)
O2—C1—C8—C7	−0.77 (18)	C12—C13—C18—C9	0.49 (15)
O1—C1—C8—C7	178.73 (10)	C14—C13—C18—C9	178.72 (10)
C18—C9—C10—C11	−0.10 (15)	C12—C13—C18—C17	−179.89 (10)
C18—C9—C10—C2	179.91 (9)	C14—C13—C18—C17	−1.65 (16)
O1—C2—C10—C11	−121.44 (10)	C16B—C17—C18—C9	160.3 (5)
C3—C2—C10—C11	121.92 (11)	C16A—C17—C18—C9	−157.81 (11)
O1—C2—C10—C9	58.55 (12)	C16B—C17—C18—C13	−19.3 (5)
C3—C2—C10—C9	−58.09 (13)	C16A—C17—C18—C13	22.56 (15)
C9—C10—C11—C12	0.51 (16)	O2—C1—O1—C2	−179.49 (9)
C2—C10—C11—C12	−179.50 (10)	C8—C1—O1—C2	0.95 (10)
C10—C11—C12—C13	−0.44 (17)	C10—C2—O1—C1	−124.41 (9)
C11—C12—C13—C18	−0.07 (17)	C3—C2—O1—C1	−0.53 (10)
